# Assessing genotoxic effects of chemotherapy agents by a robust *in vitro* assay based on mass spectrometric quantification of γ-H2AX in HepG2 cells

**DOI:** 10.3389/fphar.2024.1356753

**Published:** 2024-06-19

**Authors:** Minmin Qu, Jia Chen, Bin Xu, Qinyun Shi, Shujing Zhao, Zhaoxia Wang, Zhi Li, Bo Ma, Hua Xu, Qinong Ye, Jianwei Xie

**Affiliations:** ^1^ Department of Medical Molecular Biology, Beijing Institute of Biotechnology, Beijing, China; ^2^ Beijing Institute of Pharmacology and Toxicology, Beijing, China

**Keywords:** chemotherapy agents, genotoxicity, γ-H2AX, DNA damage repair, HepG2 cells

## Abstract

Chemotherapy has already proven widely effective in treating cancer. Chemotherapeutic agents usually include DNA damaging agents and non-DNA damaging agents. Assessing genotoxic effect is significant during chemotherapy drug development, since the ability to attack DNA is the major concern for DNA damaging agents which relates to the therapeutic effect, meanwhile genotoxicity should also be evaluated for chemotherapy agents’ safety especially for non-DNA damaging agents. However, currently applicability of *in vitro* genotoxicity assays is hampered by the fact that genotoxicity results have comparatively high false positive rates. γ-H2AX has been shown to be a bifunctional biomarker reflecting both DNA damage response and repair. Previously, we developed an *in vitro* genotoxicity assay based on γ-H2AX quantification using mass spectrometry. Here, we employed the assay to quantitatively assess the genotoxic effects of 34 classic chemotherapy agents in HepG2 cells. Results demonstrated that the evaluation of cellular γ-H2AX could be an effective approach to screen and distinguish types of action of different classes of chemotherapy agents. In addition, two crucial indexes of DNA repair kinetic curve, i.e.*, k* (speed of γ-H2AX descending) and t_50_ (time required for γ-H2AX to drop to half of the maximum value) estimated by our developed online tools were employed to further evaluate nine representative chemotherapy agents, which showed a close association with therapeutic index or carcinogenic level. The present study demonstrated that mass spectrometric quantification of γ-H2AX may be an appropriate tool to preliminarily evaluate genotoxic effects of chemotherapy agents.

## Introduction

Cancer remains one of the most dreaded diseases over the last few decades (Roy and Saikia, 2016). The incidence of cancer is extremely high, which seriously affects human health (Torre et al., 2016). At the beginning of the 20th century, Paul Ehrlich coined the word “chemotherapy,” which means to use the drugs to kill pathogenic microorganisms to treat infectious diseases (DeVita and Chu, 2008). At present, chemotherapy is very effective in cancer treatment and plays an important role in current treatment methods (Knezevic and Clarke, 2020). Broadly, chemotherapy agents presently used have been classified as: alkylating agents, antimetabolites, antitumor antibiotics, antitumor plant products, antitumor hormones and various miscellaneous agents (Masood et al., 2016; Bukowski et al., 2020). Although this classification is the one now in general usage, it is a relatively simple classification mainly based on the source, biological action, chemical reaction and other characteristics of drugs. In fact, a common trait of chemotherapy drugs is that they cause changes at the cellular level by interfering with the complex intermediate metabolism of cells or affecting cell division at the metaphase (Masood et al., 2016).

Alkylating agents, also known as bio-alkylating agents, form compounds with reactive electrophilic groups that can covalently bind to biological macromolecules within cells, thereby changing their structures and possibly destroying their functions (Fu et al., 2012). Among them, nitrogen mustard-derived DNA alkylating agents were the first antitumor drugs to achieve outstanding efficacy and remain key drugs against a variety of cancers to date (Singh et al., 2018). Although alkylating agents pose a significant threat to human health due to various toxic effects, some toxic alkylating agents are still used as chemotherapeutic agents in cancer patients (Sauter and Gillingham, 2020). Consequently, while with cancer-inducing potency, alkylating agents are still used to kill cancer cells given their DNA-damaging characteristics (Fu et al., 2012; Sauter and Gillingham, 2020). Meanwhile, the double-edged sword of their therapeutic and cytotoxic potential has received attention.

It is well known that the structural integrity of DNA is particularly important for cells to maintain normal cellular function and proliferation (Cheung-Ong et al., 2013). During the S phase of the cell cycle, the initiation of replication is inhibited when DNA is damaged, slowing DNA replication and possibly causing DNA double-strand breaks (DSBs), one of the most toxic forms of DNA damages (Waterman et al., 2020). Cell death can occur when DNA damage is too severe to be repaired (Chatterjee and Walker, 2017). So far, chemotherapy for cancer has a history of about 80 years and the idea of developing various types of antitumor drugs comes from using DNA as the target of antitumor drugs (DeVita and Chu, 2008; Sauter and Gillingham, 2020), such as antimetabolites, antitumor antibiotics and antitumor plant products. Just like alkylating agents, these agents have double-edged properties, i.e., they quickly target and hurt dividing cells, but also nonspecifically affect normal cells (DeVita and Chu, 2008).

Overall evaluation of the double-edged characteristics of DNA-damaging agents is essential for balancing the chemo-efficacy and toxicity, especially genotoxicity, unfortunately which is often obscure and still a challenge mainly due to the lack of a robust *in vitro* analysis method (Cheung-Ong et al., 2013; Motoyama et al., 2018). On the other side, there are also some chemotherapy agents, which cure cancer through other mechanisms such as cytotoxicity, hormonal mimicry or epigenetic effects (Masood et al., 2016). Given that information on genotoxicity is indispensable for evaluating the therapeutic effect and side effect of DNA damaging agents, genotoxicity data are required for chemotherapy agents.

Genotoxicity assessment is an important cutting-edge safety tool during the development of pharmaceutics, and genotoxicity assays can draw conclusions about the genotoxicity and potential carcinogenicity of chemotherapeutics (Choudhuri et al., 2021; Luan and Honma, 2022). Positive results in standard genotoxicity assays such as the Ames test, mouse lymphoma assay (MLA), and *in vitro* micronucleus (MN) assay or chromosome aberration (CA) assay are of great significance during drug development (Kirkland et al., 2005). The performance of the three most commonly used assays has been evaluated in terms of their sensitivity, specificity, and positive and negative predictivity using data from 700 rodent carcinogens and non-carcinogens (Kirkland et al., 2005). The three-test battery of mammalian cell-based assays exhibits high sensitivity but a propensity towards misleading positive results (poor specificity). The phosphorylation of histone H2AX on serine (Ser) 139 (designated as γ-H2AX), a robust biomarker of DNA damage, has emerged as a reliable tool to evaluate genotoxic effects for a long time (Kopp et al., 2019; Rahmanian et al., 2021). After the occurrence of DSBs, γ-H2AX is amplified, which reflects global genotoxic damage that could derive from diverse forms of DNA damage such as DNA adducts, DNA crosslinking, or transposition (Rahmanian et al., 2021). γ-H2AX, which is an acknowledged attractive bifunctional biomarker, is thought to be primarily related to DNA damage, but changes in γ-H2AX content also play a role in DNA repair (Qu et al., 2021).

Conventionally, γ-H2AX was extensively measured by immunology-based methods including Western blotting, immunofluorescence staining and flow cytometry (Kopp et al., 2019). Although immunoassays provide good sensitivity, their specificity is limited due to poor batch-to-batch reproducibility as well as some cross-reactivity derived from antibodies, and accurate quantification is still challenging. Previously, we developed an *in vitro* genotoxicity assay based on γ-H2AX quantification using mass spectrometry (MS). This assay has been used to assess the genotoxicity of different chemicals and demonstrates high specificity and sensitivity (Qu et al., 2020). In addition, the assay has the advantage of dynamically monitoring specific processes of DNA damage and repair caused by genotoxic chemicals. Recently, we further validate the feasibility of using this MS-based γ-H2AX *in vitro* assay to assess the potential carcinogenicity of genotoxic compounds based on a large set of compounds from the European Centre for the Validation of Alternative Methods (ECVAM) list (Qu et al., 2021), and quantitatively determined the DNA damage repair characteristics of aristolochic acids (Qu et al., 2022).

In this article, we firstly quantified γ-H2AX induced by 34 classical chemotherapy agents including DNA damaging agents and non-DNA damaging agents in HepG2 cells, based on MS. The preliminary results obtained suggest that the detection of γ-H2AX against different classes of chemotherapy agents could be an effective approach to obtain information related to the DNA-damaging efficacy of chemotherapy agents. We then proved that DSBs repair kinetics of nine tested chemotherapy agents according to γ-H2AX time-effect curves have a close association with therapeutic index and carcinogenic level, which may guide the evaluation and clinical application of chemotherapy agents.

## Materials and methods

### Materials and reagents

Thirty-four chemotherapy agents were obtained from Yuanye Bio-Technology Co., Ltd. (Shanghai, China) and their purity exceeded 98%. The identities of the 34 chemotherapy agents are shown in Table 1. All the agents were chosen from major categories (classes) based on their chemical structures and the way they act on cancer cells, representing a broad range of chemotherapy activities. Based on the concentration used in the *in vitro* genotoxicity tests reported in the literature, the maximum concentration of agents used in this study is 1 mM (Kirkland et al., 2016).

**TABLE 1:** *In vitro* genotoxicity of 34 chemotherapy agents tested by γ-H2AX MS assay.

Name of agents	CAS number	Required metabolic activation	*In vitro* assays	γ-H2AX _MEC/MS_
Alkylating agent				
Nimustine	55661-38-6	−	+	↑(0.01)
Carmustine	154-93-8	−	+	↑(0.01)
Cyclophosphamide	6055-19-2	+	+	↑(1000)
Ifosfamide	3778-73-2	+	+	↑(1000)
Antimetabolites				
5-fluorouracil	51-21-8	−	+	↑(10)
Deoxyfluridine	436349	−	+	↑(10)
Tegafur	17902-23-7	−	+	↑(10)
Carmofur	6122-45-5	−	+	↑(1)
6-mercaptopurine	50-44-2	+	+	↑(10)
Thioguanine	154-42-7	+	+	↑(10)
Hydroxyurea	127-07-1	−	+	↑(1000)
Antitumor antibiotics				
Daunorubicin	20830-81-3	−	+	↑(0.01)
Doxorubicin	25316-40-9	−	+	↑(0.01)
Pirarubicin	72496-41-4	−	+	↑(0.01)
Epirubicin hydrochloride	56390-09-1	−	+	—
Antitumor plant products				
Irinotecan	97,682-44-5	−	+	↑(0.1)
Topotecan	119413-54-6	−	+	↑(0.1)
Exatecan	171335-80-1	−	+	↑(0.01)
Etoposide	33419-42-0	−	+	↑(1)
Teniposide	29767-20-2	−	+	↑(1)
Vinorelbine	71486-22-1	−	E	—
Paclitaxel	33069-62-4	−	E	—
Vincristine	57-22-7	−	E	—
Antitumor hormones				
Tamoxifen	10540-29-1	+	−	—
Aminoglutethimide	125-84-8	−	−	—
Anastrozole	120511-73-1	−	−	—
Letrozole	112809-51-5	−	−	—
Formestane	566-48-3	−	−	—
Exemestane	107868-30-4	−	−	—
Miscellaneous agents				
Cisplatin	15663-27-1	−	+	↑(10)
Carboplatin	41575-94-4	−	+	↑(100)
Oxaliplatin	61825-94-3	−	+	↑(100)
Dacarbazine	891986	+	+	↑(100)
Mitoxantrone	65271-80-9	−	+	↑(1)

Notes: *In vitro* assays refer to the results of genotoxicity of compounds through the combination of *in vitro* mouse lymphoma, chromosomal aberration, and micronucleus tests. + tested as “positive”; − tested as “negative”; E tested as “equivocal”. Micromolar concentrations measured in the last column refer to the MEC measured by our MS method. The arrows illustrate an obvious increase in the value of γ-H2AX.

Peptide P1, ATQASQEY and peptide P2, ATQA*p*SQEY, representing the sequences of tryptic products of H2AX and γ-H2AX at its 135–142 site, and isotope-labelled peptides with ^13^C3 and ^15^N-labelled amino terminal alanine, i.e.*,* [^13^C3, ^15^N]ATQASQEY and [^13^C3, ^15^N]ATQA*p*SQEY were synthesized by Sangon Biotech Co., Ltd. (Shanghai, China). Sequencing-grade trypsin was obtained from Promega Biotech Co., Ltd. (Beijing, China). HPLC-grade acetonitrile was provided by J&K Scientific Ltd. (Beijing, China). Formic acid (FA) was purchased from Sigma-Aldrich Inc. (St. Louis, United States). Other compounds or reagents were obtained from Sinopharm Compound Reagents Co., Ltd. (Beijing, China). All reagents were of analytical reagent grade or higher.

Dulbecco’s modified Eagle’s medium (DMEM) and fetal bovine serum (FBS) were purchased from Life Technologies (Paisley, United Kingdom). A kit for performing a cell proliferation assay was obtained from Promega Biotech Co., Ltd. (Beijing, China). C18 disks were purchased from Empore, 3 M (Shanghai, China).

Ultrapure water (18.2 MΩ cm) was prepared using a Milli-Q A10 purification system from Millipore Co. (Watford, United Kingdom). Before use, all solutions were sterilized by a high-pressure sterilizer (Zhongya Co., Shanghai, China). Unless noted, stock solutions of all compounds were prepared in 100% dimethyl sulfoxide (DMSO, Sigma, St. Louis, United States).

### Cell culture and treatment

Human hepatoblastoma cells (HepG2) were cultivated in DMEM medium under standard conditions (37°C in a 5% CO_2_ atmosphere). The culturing medium was added with 10% FBS, 100 U/mL penicillin and 100 μg/mL streptomycin.

In accordance to our previous study, cells were exposed to the chemotherapeutic agent by adding a proper volume of chemotherapeutic agent stock solution to the fresh medium without serum. Controls were treated with DMSO (0.1%) in medium. Independent biological experiments with three technical replicates were conducted.

The cells were then treated with different chemotherapy agents in serum-free medium. For a dose-effect relationship experiment, cells were exposed to each agent at either of three concentrations with a 10-fold increase for 24 h. For the time-effect relationship experiment of nine tested chemotherapy agents, cells were exposed to selected chemotherapy agents at 0.5, 1, 2, 4, 8, 12, and 24 h, with the concentration of 100 μM. Independent biological experiments with three technical replicates were performed.

### Assessment of cytotoxicity

The assessment of cytotoxicity was assessed using the 3-(4,5-dimethylthiazol-2-yl)-5-(3-carboxymethoxyphenyl)-2-(4-sulfophenyl)-2H-tetrazolium (MTS) assay according to the manufacturer’s instructions (Promega), with minor modifications, after 24 h of exposure to compounds. Briefly, after exposure, a freshly prepared mixture of MTS solution was added to each well of a multiwell plate and incubated for an additional 3 h. Afterwards, cell viability was measured using a spectrofluorimeter (Synergy MX, BioTek, Winooski, United States) at 490 nm. Three independent experiments were performed for each time in six replicates, with each replicate represented by one well. The relative cytotoxicity was obtained by the ratio of the surviving cells in the treatment groups to that of control (solvent) group.

### γ-H2AX MS quantitation

The quantification of γ-H2AX was carried out according to the previous report (Qu et al., 2021). In short, the cell clumps were collected after a certain period of cell culture and histones were obtained by acid extraction. After nuclear isolation, histone extraction, trypsin digestion in the solution, and desalting, the peptide sample from the carboxy terminus of H2AX was analyzed. LC–MS/MS analysis was conducted using a QTRAP 5500 (AB Sciex, Framingham, United States) with an ACQUITY UPLC system (Waters Co., Manchester, United Kingdom). Chromatographic separation was carried out with an ACQUITY UPLC BEH C18 column (100 mm × 2.1 mm, 1.7 μm). The column temperature was maintained at 40°C. A 10 μL sample aliquot was injected for analysis. Mobile phases A and B were 0.1% FA in distilled H_2_O and acetonitrile, respectively. The elution gradient was initiated with 1% B and linearly increased to 30% B in 8 min at a flow rate of 0.25 mL/min. The eluent composition was maintained for 2 min, after which the system returned to 1% B and was re-equilibrated for 2 min. The eluates in the first 1 min were switched to waste to prevent contaminating the ion source. The electrospray ionization source was operated in positive mode using nitrogen as the nebulizing gas. All experiments were performed independently in at least triplicate.

### Statistical analysis

All data were expressed in the form of means ± standard deviation (SD). The IBM-SPSS Statistics Ver.21.0 software (IBM Corp., Armonk, NY, United States) was used for statistical analysis. Differences among treatments were evaluated by using one-way analysis of variance (ANOVA). Independent biological experiments with three technical replicates were performed. *p ≤ 0.05 was considered statistically significant, and **p ≤ 0.01 was considered highly significant.

## Results and disscussion

### Cytotoxicity evaluation of 34 chemotherapy agents in HepG2 cell line

**FIGURE 1:**
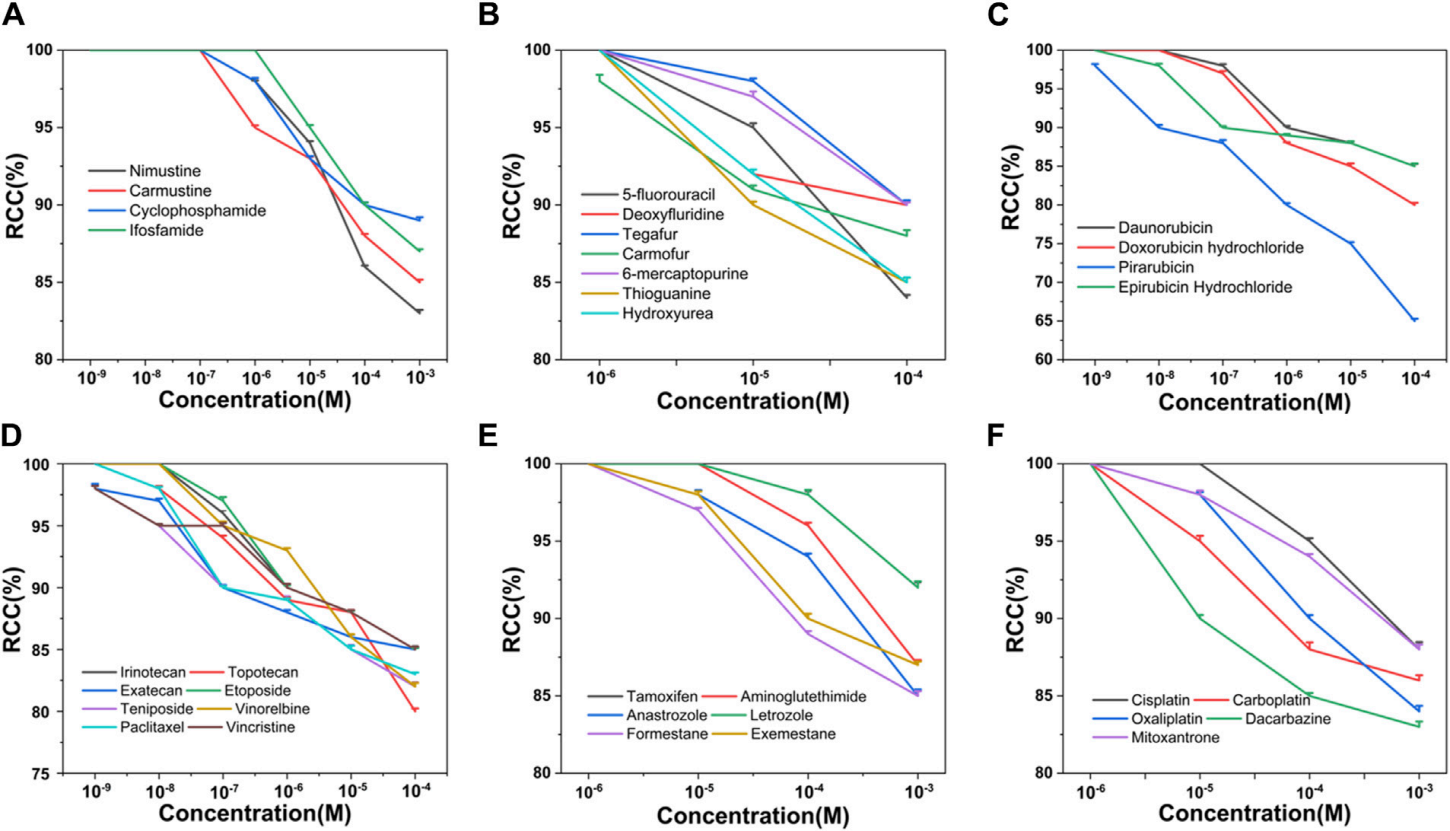
Cytotoxicity of 34 chemotherapy agents in HepG2 cells, including alkylating agents **(A)**, antimetabolites **(B)**, antitumor antibiotics **(C)**, antitumor plant products **(D)**, antitumor hormones **(E)** and various miscellaneous agents **(F)**. The value of RCC indicates the cytotoxicity. Each value was expressed in the form of mean ± SD (*n* ≥ 3).

Based on the criteria of positive genotoxicity (Qu et al., 2021), a compound that resulted in a 1.5-fold increase in the value of γ-H2AX and produced a level of cytotoxicity below 50% relative to the control group, is considered to be genotoxic. To this end, we firstly examined the cytotoxicity of 34 chemotherapy agents, all of which were already well classified in terms of diverse characteristics. These tested chemotherapy agents were serially diluted for exposure to check the effects on cell viability. Relative cell count (RCC; % control) results were obtained by the MTS assay. As shown in Figure 1, the values of RCC for all chemotherapy agents were higher than 50% in HepG2 cells within our chosen exposure concentration range.

### γ-H2AX tested results for alkylating agents

**FIGURE 2:**
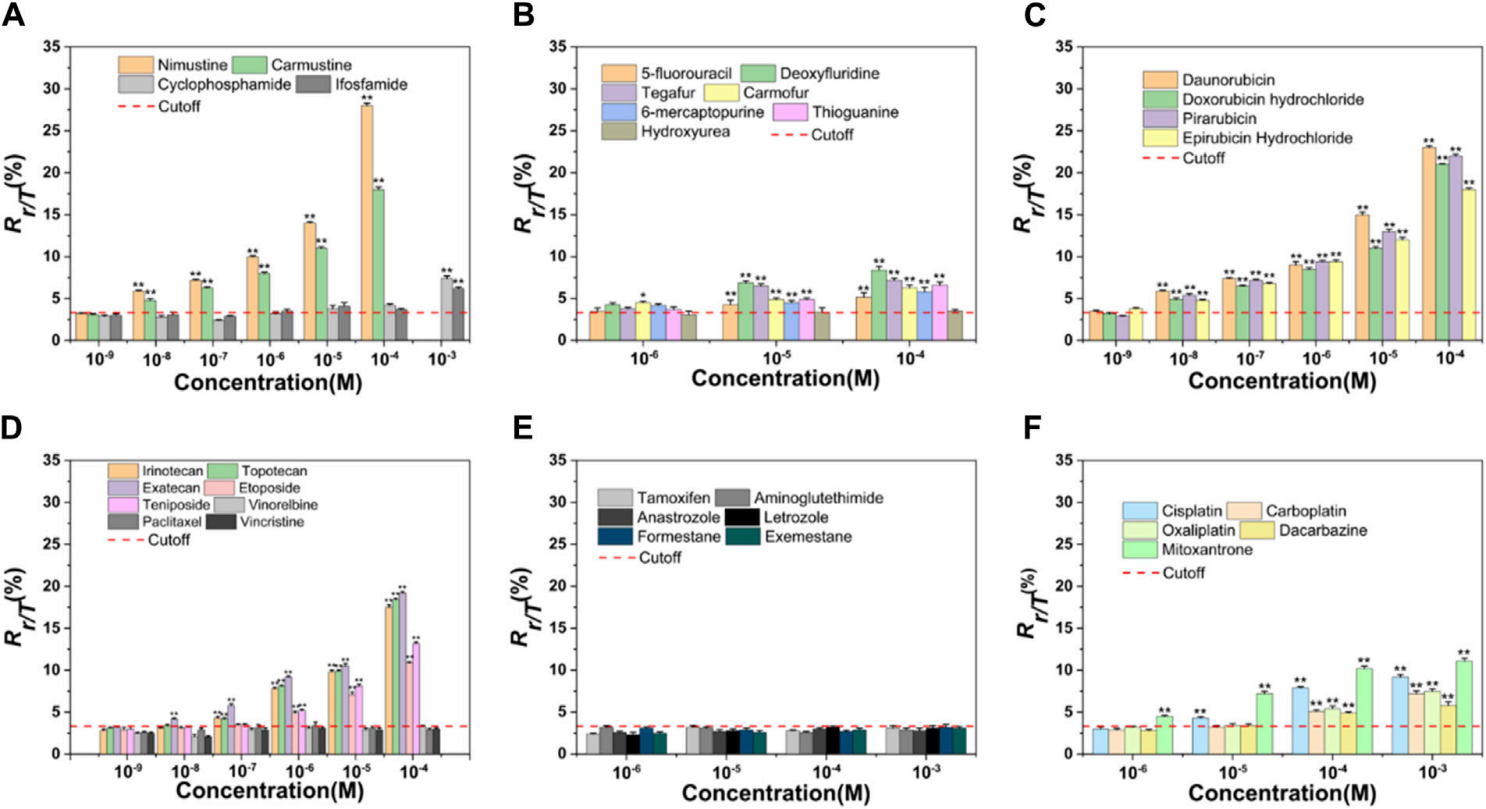
Dose-effect relationships of γ-H2AX after HepG2 cells were exposed to 34 chemotherapy agents for 24 h, including alkylating agents **(A)**, antimetabolites **(B)**, antitumor antibiotics **(C)**, antitumor plant products **(D)**, antitumor hormones **(E)** and various miscellaneous agents **(F)**. The horizontal axis represents different concentrations and the vertical axis represents the proportion of the number of phosphorylated peptides to the total number of peptides (R_γ-H2AX/Total H2AX_, briefly, *R*_*γ/T*_) in a cell. The differences between chemotherapy agents treated groups and negative control are obvious (*n* ≥ 3, mean ± SD; *p ≤ 0.05, **p ≤ 0.01). Red dashed line represents the solvent control value of HepG2 cells.

Nitrogen mustard ushered in a new era in cancer chemotherapy in 1942 (Chabner and Roberts, 2005). At the molecular level, nitrogen mustard produces an intermediate called an “aziridiniumion” that is highly reactive against DNA in both tumor and normal cells, leading to serious side effects and therapeutic implications (Singh et al., 2018). This class of valuable alkylating agents can bind covalently to DNA in an essentially irreversible manner, resulting in major changes in DNA structure and function (Misiak et al., 2016). To improve efficacy and enhance specificity for tumor cells, various nitrogen mustard derivatives have been developed (DeVita and Chu, 2008), including DNA alkylators nimustine, carmustine, cyclophosphamide, and ifosfamide, all of which are widely used in clinical treatment. We tested the four alkylating agents in HepG2 cells for γ-H2AX biomarker. Table 1 and Figure 2A showed the results.

As chloroethylnitrosoureas derivatives, nimustine and carmustine are typical chloroethylating agents which can be used in tumor chemotherapy, especially brain tumors due to the capacity to get over the blood-brain barrier (Nikolova et al., 2012; Nikolova et al., 2017). These two reagents can combine with guanine N1 on one DNA strand and cytosine N3 on the other strand to form inter-strand crosslinks and prevent DNA replication (Drabløs et al., 2004; Yamada et al., 2019). However, carmustine was reported to be six to eight times less cytotoxic than nimustine in cell lines (Büch and Zeller, 2002). In our result, nimustine and carmustine induced significant phosphorylation of H2AX at Ser 139 with a minimum effective concentration (MEC) of 0.01 µM. Nimustine-induced γ-H2AX amount was always greater than that induced by carmustine at the same molar concentration, which was consistent with the report from Nikolova et al. (2017), where authors indicated that nimustine had a higher potency for inducing deoxyribonucleic acid inter-strand crosslinks than carmustine. Our result indicated that the genotoxicity of nimustine and carmustine can be preliminarily obtained by comparing the γ-H2AX values, even if their MEC values were same, which may be an effective approach to obtain related information on efficacy of alkylating agents.

During the development of nitrogen mustard derivatives, developing alkylating agents which exhibit genotoxic ability after enzymatic degradation is a route to pursue selectivity (World Health Organization, 1962). Both cyclophosphamide and ifosfamide are examples of this, and the two agents are highly stable and need to be activated by liver microsomal enzymes metabolism. After the two agents are distributed throughout the body, they spontaneously degrade at the tumor site to form their own cytotoxic substances, namely, phosphoramide mustard and ifophosphamide mustard (Mulder et al., 2015). These cytotoxic species will alkylate DNA, forming inter-strand crosslinks which ultimately inhibit DNA synthesis (Fresneau et al., 2017). As observed in Figure 2A, the highest concentration of cyclophosphamide and ifosfamide tested (1 mM) was genotoxic. The results for cyclophosphamide and ifosfamide, which share a genotoxic mode of action related to a specific biotransformation process, indicated a good sensitivity for γ-H2AX MS analysis (Wu et al., 2017). Although ifosfamide and cyclophosphamide are closely related (Fresneau et al., 2017), structural changes in ifosfamide result in a reduction in liver activation, which in turn reduces efficacy. In our result, the value of γ-H2AX induced by ifosfamide was lower than that of cyclophosphamide under the same exposure concentration of 1 mM.

### γ-H2AX tested results for antimetabolites

Antimetabolites are a class of antineoplastic agents that disrupt DNA replication. Most antimetabolites belong to the category of chain-terminating nucleoside analogs which interfere with subsequent steps of DNA biosynthesis through competitive inhibition (Masood et al., 2016). Among them, pyrimidine analogs and purine analogs are widely used. During the S phase of the cell cycle, purine and pyrimidine analogs are able to incorporate into DNA and prevent nucleotide addition, leading to DNA replication failure (Lansiaux, 2011).

Seven agents with antimetabolite mechanism of action were tested: 5-fluorouracil (5-FU), deoxyfluridine, tegafur, carmofur, 6-mercaptopurine (6-MP), thioguanine (TG), and hydroxyurea (Table 1). Since the action mechanism of these agents were mainly metabolite-related (Masood et al., 2016), antimetabolites would not cause large change in the value of γ-H2AX within the selected concentration range, as shown in Figure 2B. Of these, carmofur with MEC of 1 µM was the only antimetabolite which induced an increase of γ-H2AX in a dose-dependent manner at the three tested concentrations, with no apparent cytotoxicity. 5-FU, deoxyfluridine, tegafur, 6-MP, and TG had a MEC of 10 µM. As previously mentioned, hydroxyurea suppresses pyrimidine biosynthesis by inhibiting the enzyme ribonucleotide reductase thus exhibiting a MEC of 1 mM in γ-H2AX MS assay (Qu et al., 2020).

5-FU is a naturally occurring analogue of pyrimidine uracil and is metabolized in the same way as uracil. Due to the fact that most antimetabolites have poor selectivity and are toxic to normal tissues that proliferate rapidly, such as bone marrow, gastrointestinal mucosa and skin (Ciaffaglione et al., 2021), many derivatives of 5-FU, including deoxyfluridine, tegafur and carmofur, have been devised to improve the topical delivery and reduce the side effects. As shown in Figure 2B, at equimolar concentration, the values of γ-H2AX induced by 5-FU were always the lowest, in support of that, the therapeutic indexes of three derivative agents are higher than that of 5-FU (Hashimoto et al., 2020).

6-MP and TG belong to purine nucleoside analogues. Studies have shown that both agents undergo broad metabolism prior to incorporation into DNA to induce cytotoxicity (Elgemeie, 2003; Coulthard and Hogarth, 2005). As observed in Figures 1B, 2B, the cytotoxicity and genotoxicity of TG were stronger than that of 6-MP in HepG2 cell line at equimolar concentration. Since TG can be directly converted to thioguanine nucleotides, avoiding a few enzymatic checkpoints in 6-MP metabolism, TG exhibits higher toxicity (Lennard et al., 1993). Additionally, Adamson et al. has demonstrated that TG has higher cytotoxicity than 6-MP in diverse cell lines (Adamson et al., 1994).

### γ-H2AX tested results for antitumor antibiotics

Clinically, anthracyclines are a class of natural antibiotics among the most effective antineoplastic drugs, acting against nearly all cancer types (Martins-Teixeira and Carvalho, 2020). Anthracyclines share planar aromatic rings that can enter and form stacking relations between near DNAs to stabilize, harden, prolong and relax the DNA double helix (Bauer and Vinograd, 1970; Canals et al., 2005; Martins-Teixeira and Carvalho, 2020). Anthracyclines exert the anticancer action via intercalation into the double helix of DNA, binding to topoisomerase II, and generation of reactive oxygen species (Meredith and Dass, 2016; Martins-Teixeira and Carvalho, 2020). Four anthracyclines (daunorubicin, doxorubicin, pirarubicin, epirubicin hydrochloride) with an antitumor antibiotic effect were tested. These selected four antitumor antibiotics had the same MEC (0.01 µM) in the cell line used. For all these four agents, we found that they induced γ-H2AX/H2AX_total_ (R_γ/T_) value was as high as 25% at the higher concentration, which might explain that anthracyclines are the most potent anti-cancer chemotherapeutics (Weiss, 1992; Martins-Teixeira and Carvalho, 2020).

Both doxorubicin and pirarubicin belong to second-generation antibiotics. Because pirarubicin was more efficient for entering cells (Miller and Salewski, 1994), the γ-H2AX level caused by pirarubicin showed a slight tendency to be higher than that of doxorubicin. According to the literature, second-generation analogues like doxorubicin and pirarubicin exhibit improvements in their therapeutic indexes compared with the first-generation antibiotic daunorubicin (Minotti et al., 2004). Here, the γ-H2AX level they induced was not as high as daunorubicin. This may be due to the broad antitumor spectrum of second-generation antibiotics.

Epirubicin hydrochloride, an isomer of doxorubicin (Minotti et al., 2004), is as effective as doxorubicin. As observed in Figure 2C, these two agents induced almost the same level of γ-H2AX at equimolar concentration.

### γ-H2AX tested results for antitumor plant products

The effect of a set of eight antitumor plant products (Table 1) on γ-H2AX in the HepG2 cell line were evaluated. Table 1 and Figure 2D showed the results. Irinotecan and topotecan had MECs of 0.1 µM, exatecan had a MEC of 0.01 µM, etoposide and teniposide had a MEC of 1 µM. Our previous work showed that aneugens induced γ-H2AX less than 1.5-fold compared to controls in HepG2 cells, identified as a negative test result (Qu et al., 2020). Consistent with our previous report, here three agents with an aneugen genotoxicity, vinorelbine, paclitaxel and vincristine, were detected as with no variations of γ-H2AX in HepG2 cells.

It is well known that camptothecin plays a crucial role in clinical cancer therapy, and many pharmaceutical researchers are working on developing its derivatives (Soepenberg et al., 2003). As derivatives of camptothecin, topotecan and exatecan have been approved by the FDA and used in clinical practice (Zhu et al., 2018). With no doubt, the anticancer activities of camptothecin derivatives emerge from their potent and specific inhibition of the ubiquitous DNA-manipulating enzymes, DNA topoisomerases. DNA topoisomerases are inherent enzymes existing in all nucleated cells with two major topoisomerase forms: the type I enzyme which catalyses the change of topological isomerism of DNA replication by forming a short single strand cleavage and type II enzyme which changes the topological state of DNA by causing the break of the transient double stranded enzyme bridge (Wang, 1996). These enzymes are related to the adjustment of DNA topology and are required for the maintenance of the completeness of DNA structure during DNA metabolism (Champoux, 2001).

In our result, the MEC for exatecan causing H2AX significant phosphorylation was lower than that for irinotecan and topotecan, thus, the genotoxicity of exatecan seems to be stronger. It is reported that exatecan is a totally synthetic analogue that does not require enzymatic activation like some of the other prodrugs such as irinotecan (Soepenberg et al., 2003). Here, exatecan was also evidenced to be a more potent inhibitor of topoisomerase I than irinotecan and topotecan.

As semisynthetic derivatives of podophyllotoxin, etoposide and teniposide are growingly used in cancer treatment. Etoposide is one of the topoisomerase II poisons, which stabilizes topoisomerase II on DNA, leading to a toxic DNA-topoisomerase II covalent complex. Teniposide mainly block DNA synthesis by inhibiting the action of DNA topoisomerase II (Holthuis, 1988). Our results indicated that γ-H2AX induced by teniposide always showed a slightly higher trend than that of etoposide at different concentration administration groups, which is in line with the report of Clark et al., that teniposide was more effective in generating the DNA damage and cytotoxicity (Clark and Slevin, 1987). In addition, *in vitro* studies have demonstrated that the topoisomerase I inhibitors were more mutagenic relative to topoisomerase II inhibitors (Soepenberg et al., 2003). As observed in Figure 2D, the value of γ-H2AX caused by topoisomerase I inhibitors was always higher than that of topoisomerase II-inhibitors at equimolar concentration.

Contrary to the above five topoisomerase inhibitors, three microtubule inhibitors did not increase the phosphorylation value of H2AX in cells. During the metaphase of the cell cycle, microtubule inhibitors function by disturbing cell division, which does not affect DNA synthesis (Yamada and Gorbsky, 2006). These changes may result in aneuploidy in daughter cells and cell cycle dysregulation etc., instead of real DNA damage (Aardema et al., 1998). Numerous studies have described the effects of microtubule inhibitors on H2AX phosphorylation. In our previous study, microtubule inhibitors did not cause H2AX phosphorylation in HepG2 and HeLa cells (Qu et al., 2020). Nonetheless, based on an in-cell Western technique, researchers (Khoury et al., 2013) found that microtubule inhibitors led to changes of γ-H2AX level in HepG2 cells, which may be a false positive result because the concentrations used in their experiment were higher than in ours. Although the result needs to be verified by performing the MS of γ-H2AX in a larger number of microtubule inhibitors, our results support that the γ-H2AX quantitation by MS analysis may be more specific in genotoxicity assessment.

### γ-H2AX tested results for antitumor hormones

Oral hormonal agents have been used to treat cancer for many years (Masood et al., 2016). In our work, we chose six antitumor hormones (tamoxifen, aminoglutethimide, anastrozole, letrozole, formestane and exemestane) and monitored the γ-H2AX levels in HepG2 cells. As shown in Figure 2E, none of the selected antitumor hormones exhibited genotoxicity in the HepG2 cell line, even when the tested concentration of agents was 1 mM.

Many breast cancers require estrogen to maintain growth, and they regress without these hormones (Gompel, 2019). As a widely used endocrine agent, tamoxifen has been the first-line treatment for postmenopausal metastatic breast cancer. Tamoxifen is a selective estrogen receptor (ER) regulator which competes with estradiol for the ER and forms a stable complex with it, thereby inhibiting the growth and development of cancer cells (Jordan and Dowse, 1976). Recently, agents acting differently from tamoxifen by inhibiting aromatase and converting androgens into estrogens have been developed (Gradishar, 2004). These reagents can be essentially divided into two classes: type I steroids, which compete at the substrate-binding site, and type II nonsteroids, which interfere with the aromatase (Kharb et al., 2020).

Formestane and exemestane belong to type I steroids, whereas aminoglutethimide, anastrozole and letrozole are type II nonsteroids. Obviously, these agents act primarily on non-DNA targets and they do not damage DNA (Masood et al., 2016). γ-H2AX is a typical marker closely associated with DNA damage, which promotes related repair proteins to the damage sites in the course of DNA damage repair (Rahmanian et al., 2021). Hence, it’s not difficult to understand why these antitumor hormones do not induce a significant γ-H2AX induction.

### γ-H2AX tested results for miscellaneous agents

Agents that do not belong to the above mentioned types or whose mechanisms of action without full clarification are classified as miscellaneous agents. In our work, we selected five miscellaneous agents. As shown in Figure 2F, the MECs of cisplatin and mitoxantrone were 10 and 1 μM, and those of carboplatin, oxaliplatin and dacarbazine were 100 µM.

The development of platinum-based agents is of great significance to the research of antitumor drugs. Studies have shown that cisplatin could induce DNA damage, hinder the generation of DNA, mRNA and protein, prevent DNA replication, and ultimately result in the occurrence of apoptosis or necrosis (Rosenberg et al., 1965; Ghosh, 2019). Unfortunately, cisplatin has not shown its greatest potential in clinical use due to side effects and resistance. For this reason, drugs including carboplatin and oxaliplatin have been developed that act in a similar way of cisplatin but with different pharmacological properties and synergistic effect on different tumors (Monneret, 2011). Compared to cisplatin, carboplatin requires a higher dosage for efficacy and oxaliplatin creates fewer crosslinks per base (Dilruba and Kalayda, 2016).

The main mode of action of platinum-based analogues is to induce DNA damage. On the other hand, because of the genotoxicity, such drugs will in turn lead to tumor formation (Monneret, 2011; Dilruba and Kalayda, 2016). We observed that the value of γ-H2AX induced by cisplatin was the highest among the selected three platinum agents. This may be attributed to the fact that the side effects of platinum chemotherapy drugs are reduced with the development of platinum drugs from generation to generation, namely, carboplatin and oxaliplatin have decreased side effects (Dilruba and Kalayda, 2016).

Dacarbazine is an antitumor drug independent of cell cycle, which could exert an alkylation effect or interfere with purine biosynthesis (Al-Badr and Alodhaib, 2016). The therapeutic efficacy of dacarbazine is low due to the consequence of rapid removal of DNA lesions by repair systems (Koprowska and Czyż, 2011). Since it needs to be metabolized and activated in the liver to become an active metabolite, dacarbazine would not induce significant phosphorylation of H2AX until the concentration reaches 100 µM.

Mitoxantrone is a synthetic anthraquinone and a recognized antitumor drug. It embeds into DNA to inhibit topoisomerase II enzyme, thus preventing the connection of DNA strands and delaying the progress of cell cycle. Although mitoxantrone has been identified as a DNA topoisomerase II poison in mammalian cells, studies have determined that the drug interacts with a wider range of biological macromolecules in covalent and non-covalent ways (Scott and Figgitt, 2004). The MEC of mitoxantrone was 1 μM, which may be due to its extensive toxicity other than just as a topoisomerase II poison.

### Dynamic profiles of γ-H2AX in HepG2 cell line treated with nine representative chemotherapy agents

**FIGURE 3:**
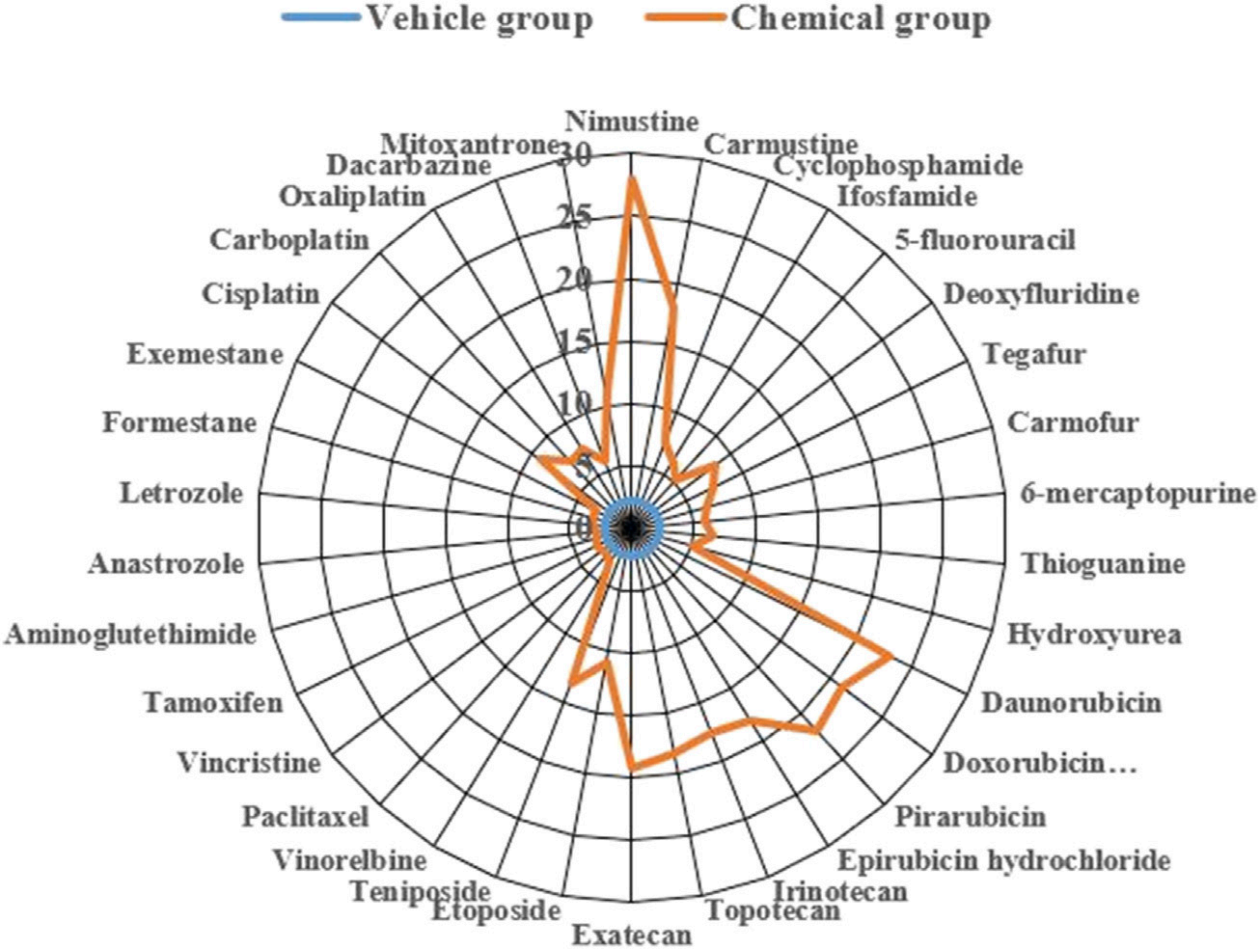
Radar plot of data for 34 chemotherapy agents with different mode of actions on γ-H2AX in HepG2 cells. Orange line represents the chemical group and blue line represents the solvent control group.

**FIGURE 4:**
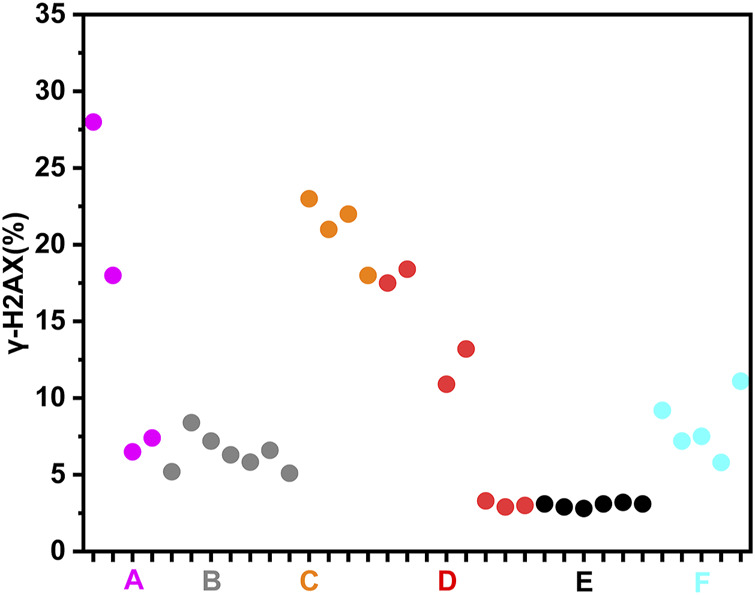
Scatterplot of data for chemotherapy agents on γ-H2AX in HepG2 cells. “A” refers to alkylating agents; “B” refers to antimetabolites; “C” refers to antitumor antibiotics; “D” refers to antitumor plant products; “E” refers to antitumor hormones; “F” refers to miscellaneous agents.

To more clearly demonstrate effects of chemotherapy agents on γ-H2AX, the values of γ-H2AX in HepG2 cell line caused by 34 chemotherapy agents were shown in Supplementary Table S1. The data were further plotted as radar chart and scatterplot. As observed in Figures 3, 4, values of R_γ/T_ induced by different classes of chemotherapy agents were varying. Agents with diverse classification have obviously different values of γ-H2AX. Additionally, agents function mainly on non-DNA targets, like antitumor hormones and aneugens in antitumor plant products, could be explicitly distinguished from DNA-targeted agents by the radar chart and scatterplot.

**FIGURE 5:**
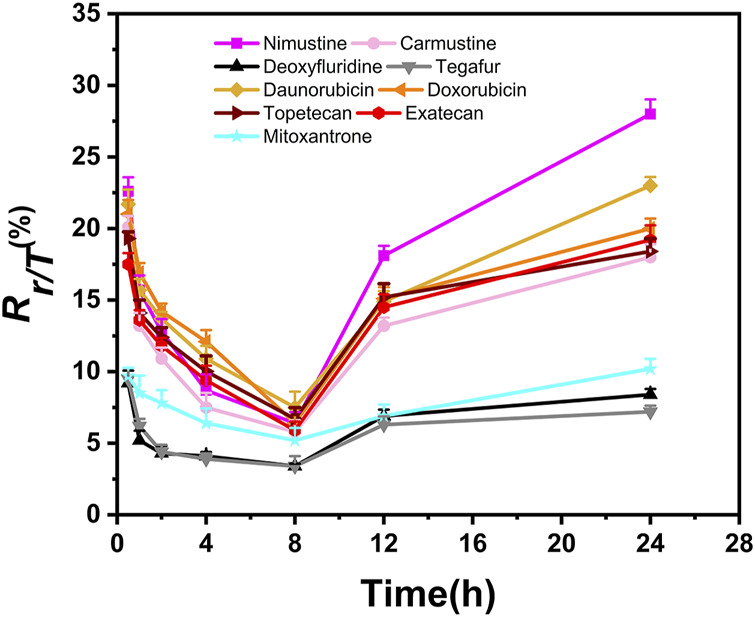
Time-dependent curves of γ-H2AX after HepG2 cells exposed to nine selected chemotherapy agents at the concentration of 100 μM. Seven time points were checked within 24 h, including 0.5, 1, 2, 4, 8, 12 and 24 h (*n* ≥ 3, mean ± SD).

In addition, we aimed to further obtain the specific profiles related to DNA damage repair induced by chemotherapy agents. To this end, we selected nine representative chemotherapy agents which induce relatively large γ-H2AX value in their respective categories, and thus investigated time effect relationship of these nine agents within 24 h. As shown in Figure 5, we found that the shapes of time effect curves for the nine representative chemotherapy agents were similar. A sharp drop was observed after 0.5 h of treatment and followed by a slow drop after 2 h. The level of γ-H2AX in a cell for the nine agents slowly decreased to the lowest value at the time of 8 h. After that, the proportion of γ-H2AX increased from 8 to 24 h.

Recently, studies have demonstrated that chemical genotoxicity has a close association with the DNA repair capacity after the exposure (Lee et al., 2019). To investigate the DNA repair ability, the data of γ-H2AX time effect after cells exposure to chemicals have been used to simulate DNA repair kinetics, and a network server (http://ccb1.bmi.ac.cn:81/shiny-server/sample-apps/prediction) was correspondingly developed to calculate two crucial indexes reflecting DNA damage and repair, that is, *k* (speed of γ-H2AX descending) and t_50_ (time required for γ-H2AX to drop to half of the maximum value) (Qu et al., 2021). Here, we estimated *k* and t_50_ after exposure to nine representative chemotherapy agents based on the 0.5–8 h γ-H2AX time-dependent kinetics (Figure 5) and then obtained these parameters to describe each agent DSB repair capacity using the functions *optim and optimize* in R language.

As shown in Table 2, we found *k* and t_50_ values were close among the selected chemotherapy drugs, except for two antimetabolites and mitoxantrone. Typically, due to the low therapeutic index, antimetabolites are used at higher clinical doses than other chemotherapy agents (Lansiaux., 2011). For example, the clinical doses of deoxyfluridine and tegafur are around 15–20 mg/kg. The relatively large *k* and relatively small t_50_ values of two antimetabolites reflect repair of DNA damage induced by antimetabolites was comparatively easier than that induced by other chemotherapy agents. Moreover, the values of γ-H2AX induced by deoxyfluridine (8.4) and tegafur (7.2) were both low at 100 μM of exposure concentration. In short, the values of *k*, t_50_ and γ-H2AX for antimetabolites indicated that antimetabolites cause weak DNA damage, that is, low genotoxicity, which may also be in part consistent with their low therapeutic indexes (Lansiaux, 2011; Masood et al., 2016). As for mitoxantrone, it induces DNA damage via a broader range of biological mechanism of action (Scott and Figgitt, 2004). Therefore, the *k* of mitoxantrone was smaller and the t_50_ of mitoxantrone was longer. In addition, similar to our previously reported work, *k* and t_50_ values varied with the carcinogenic grades of agents. Daunorubicin and doxorubicin belong to the 2B and 2A groups of the IARC classification, respectively. As shown in Table 2, the *k* of daunorubicin was greater than that of doxorubicin, and the t_50_ of daunorubicin was smaller, which support DNA damage related repair induced by doxorubicin was comparatively harder than that induced by daunorubicin.

**TABLE 2:** *k* and t_50_ values of the selected nine chemotherapy agents.

Name of chemicals	*k*	t_50_ (h)	Class
Nimustine	0.160	2.465	Alkylating agents
Carmustine	0.169	2.298	
Deoxyfluridine	4.157	1.201	Antimetabolites
Tegafur	3.015	1.442	
Daunorubicin	0.139	3.344	Antitumor antibiotics
Doxorubicin	0.088	4.269	
Topotecan	0.132	3.545	Antitumor plant products
Exatecan	0.114	3.925	
Mitoxantrone	0.111	7.999	Miscellaneous agents

## Conclusion

In summary, the γ-H2AX MS technique in HepG2 cell line seems to be a proper way to evaluate genotoxicity caused by chemotherapy agents, enabling preliminary classification of the agents and providing preprimary reference data for therapeutic effect assessment and safety evaluation. The quantification of γ-H2AX is extremely easy, gives highly specific and repeatable results, and provides a guided evaluation of chemotherapy agents, which is a potential *in vitro* assay that may eventually reduce the number of animals required for genotoxicity assessment experiments of chemotherapy agents. Future work is needed to be expanded to complementary cell lines with different metabolic activities to further confirm the feasibility of this method in assessing genotoxic effects of chemotherapy agents which may give extra important information about metabolic activation involved in genotoxicity induction.

## Data Availability

The raw data supporting the conclusions of this article will be made available by the authors, without undue reservation.
