# Supplementary material for: Assessing genotoxic effects of chemotherapy agents by a robust in vitro assay based on mass spectrometric quantification of γ-H2AX in HepG2 cells
Source: Front Pharmacol. 2024 Jun 19;15:1356753. doi: 10.3389/fphar.2024.1356753 (PMC11219945; doi:10.3389/fphar.2024.1356753)
Supplement: Supplementary file 1 [file Table1.doc]

**Table** **S1** The maximum γ-H2AX value measured for the corresponding compound within the selected concentration range

| **Name of Agents** | **CAS number** | **Required Metabolic Activation** | **γ-H2AX** |
| --- | --- | --- | --- |
| **Alkylating agent** |  |  |  |
| Nimustine | 55661-38-6 | **-** | 28 |
| Carmustine | 154-93-8 | **-** | 18 |
| Cyclophosphamide | 6055-19-2 | + | 7.4 |
| Ifosfamide | 3778-73-2 | + | 6.2 |
| **Antimetabolites** |  |  |  |
| 5-fluorouracil | 51-21-8 | **-** | 5.2 |
| Deoxyfluridine | 436349 | **-** | 8.4 |
| Tegafur | 17902-23-7 | **-** | 7.2 |
| Carmofur | 61422-45-5 | **-** | 6.3 |
| 6-mercaptopurine | 50-44-2 | + | 5.82 |
| Thioguanine | 154-42-7 | + | 6.6 |
| Hydroxyurea | 127-07-1 | **-** | 5.1 |
| **Antitumor antibiotics** |  |  |  |
| Daunorubicin | 20830-81-3 | **-** | 23 |
| Doxorubicin | 25316-40-9 | **-** | 21 |
| Pirarubicin | 72496-41-4 | **-** | 22 |
| Epirubicin hydrochloride | 56390-09-1 | **-** | 18 |
| **Antitumor plant products** |  |  |  |
| Irinotecan | 97682-44-5 | **-** | 17.5 |
| Topotecan | 119413-54-6 | **-** | 18.4 |
| Exatecan | 171335-80-1 | **-** | 19.2 |
| Etoposide | 33419-42-0 | **-** | 10.9 |
| Teniposide | 29767-20-2 | **-** | 13.2 |
| Vinorelbine | 71486-22-1 | **-** | 3.3 |
| Paclitaxel | 33069-62-4 | **-** | 2.9 |
| Vincristine | 57-22-7 | **-** | 3 |
| **Antitumor hormones** |  |  |  |
| Tamoxifen | 10540-29-1 | + | 3.1 |
| Aminoglutethimide | 125-84-8 | **-** | 2.9 |
| Anastrozole | 120511-73-1 | **-** | 2.8 |
| Letrozole | 112809-51-5 | **-** | 3.1 |
| Formestane | 566-48-3 | **-** | 3.2 |
| Exemestane | 107868-30-4 | **-** | 3.1 |
| **Miscellaneous agents** |  |  |  |
| Cisplatin | 15663-27-1 | **-** | 9.2 |
| Carboplatin | 41575-94-4 | **-** | 7.2 |
| Oxaliplatin | 61825-94-3 | **-** | 7.51 |
| Dacarbazine | 891986 | + | 5.8 |
| Mitoxantrone | 65271-80-9 | **-** | 11.1 |

Notes: + tested as “positive”; − tested as “negative”; the values in the last column are the maximum γ-H2AX value measured for the corresponding compound within the selected concentration range.
